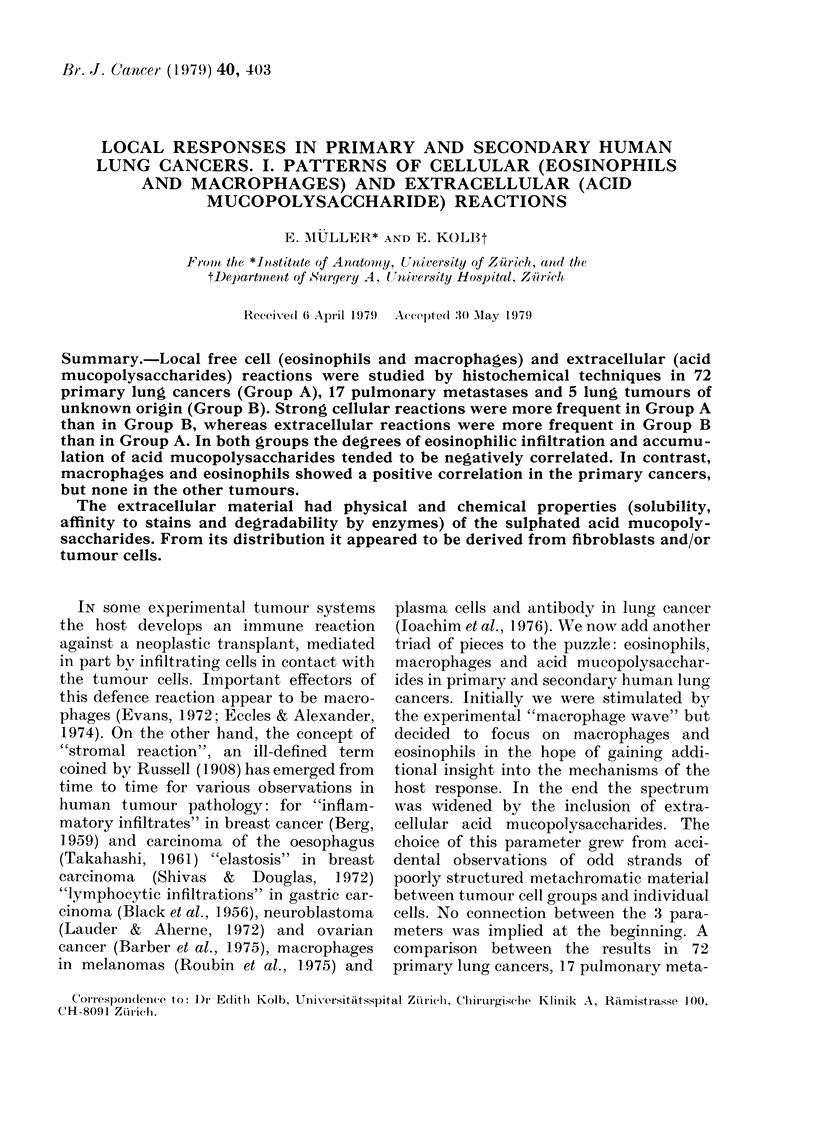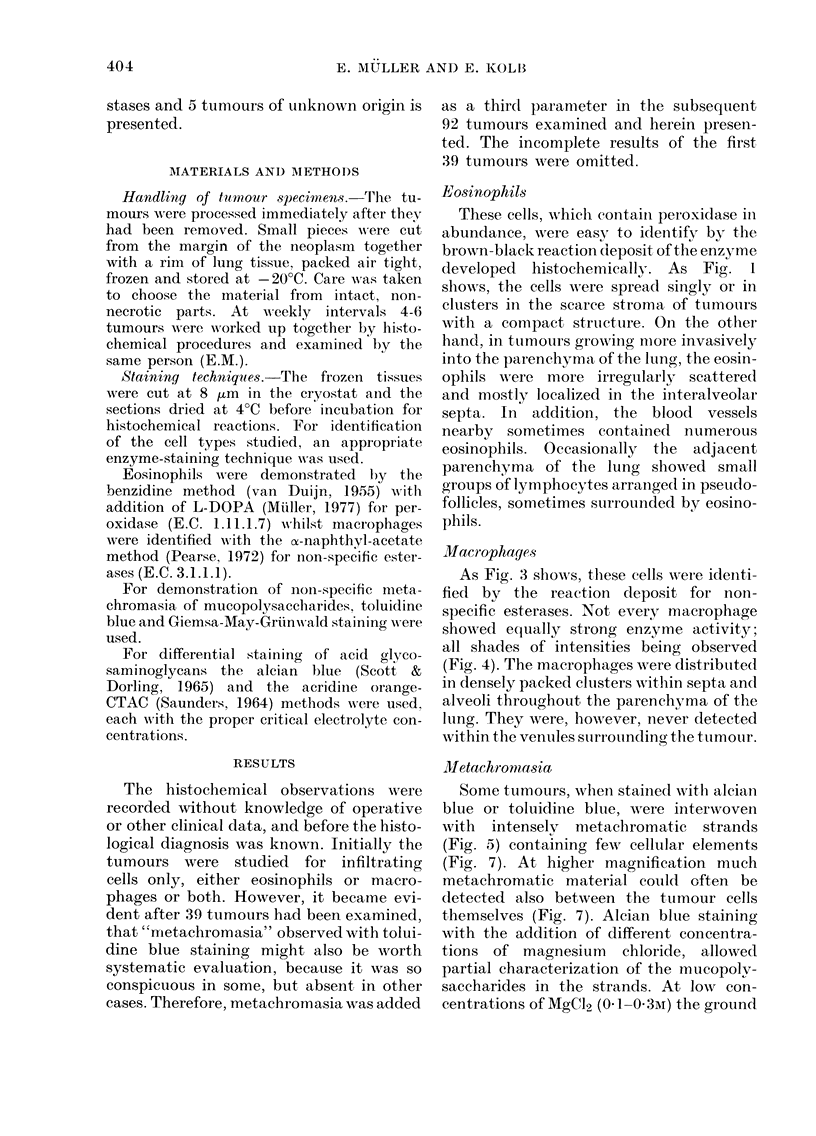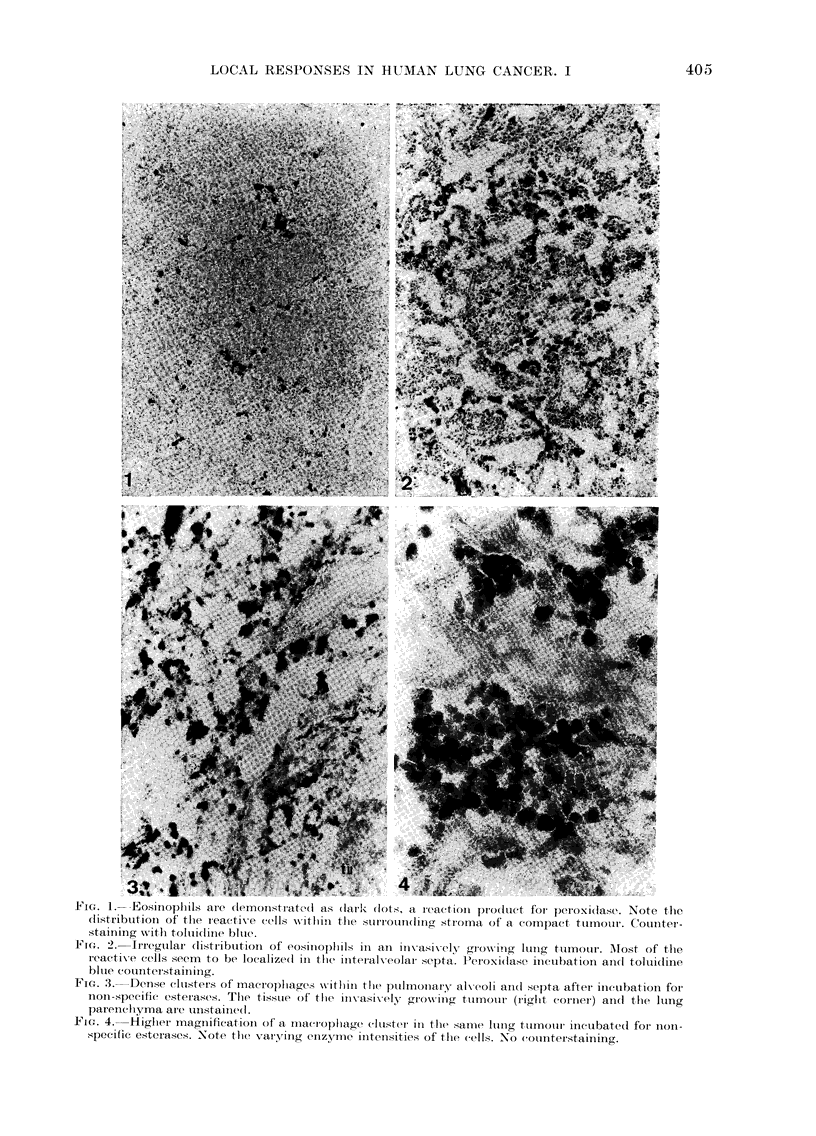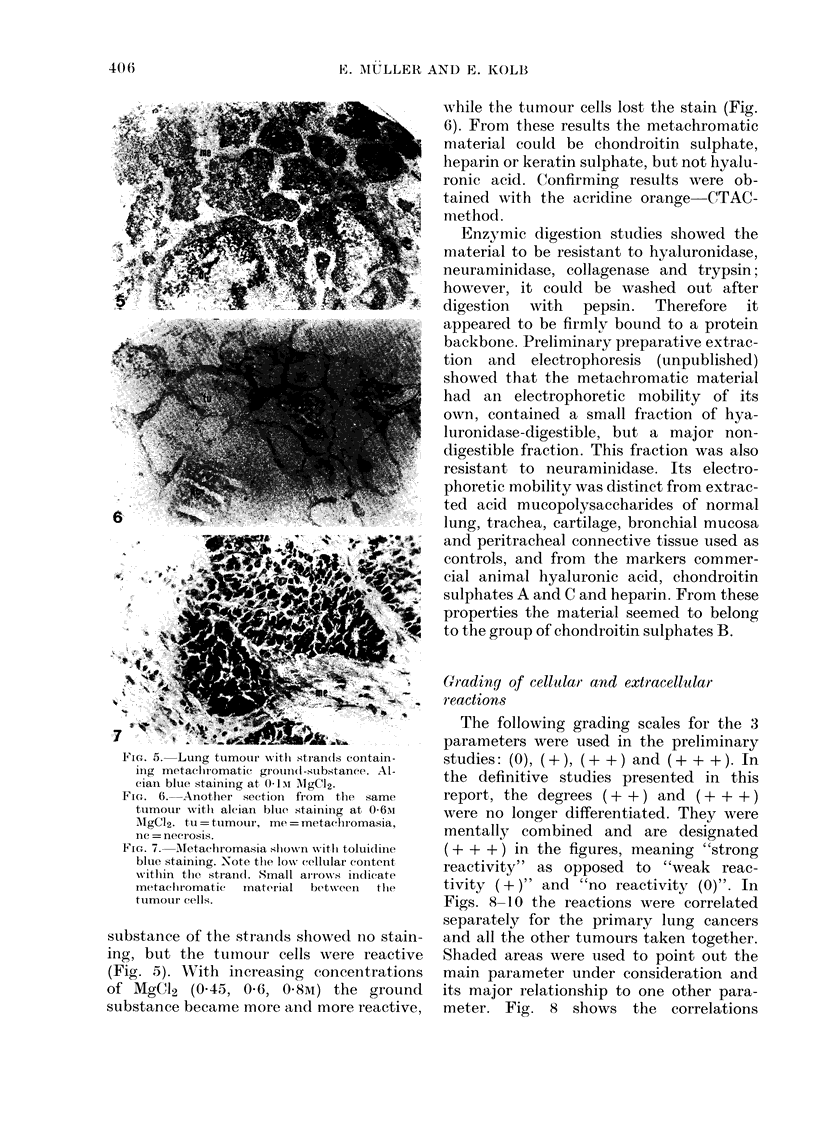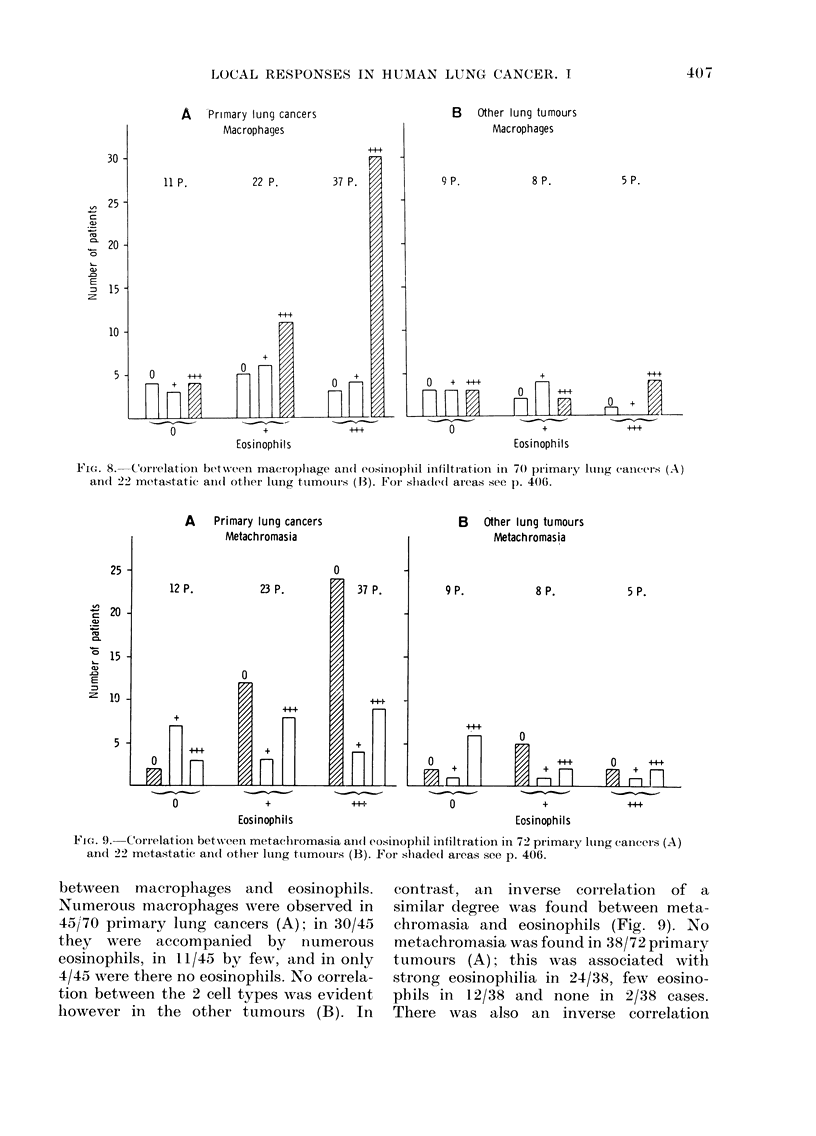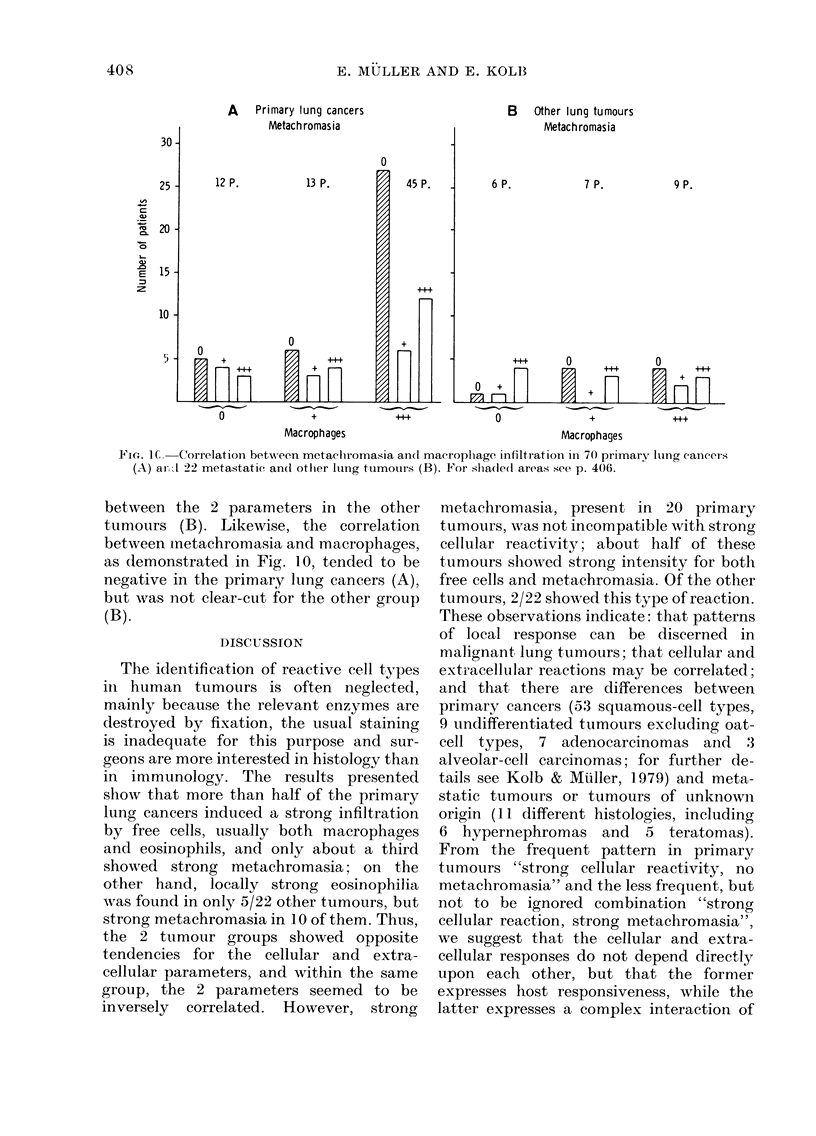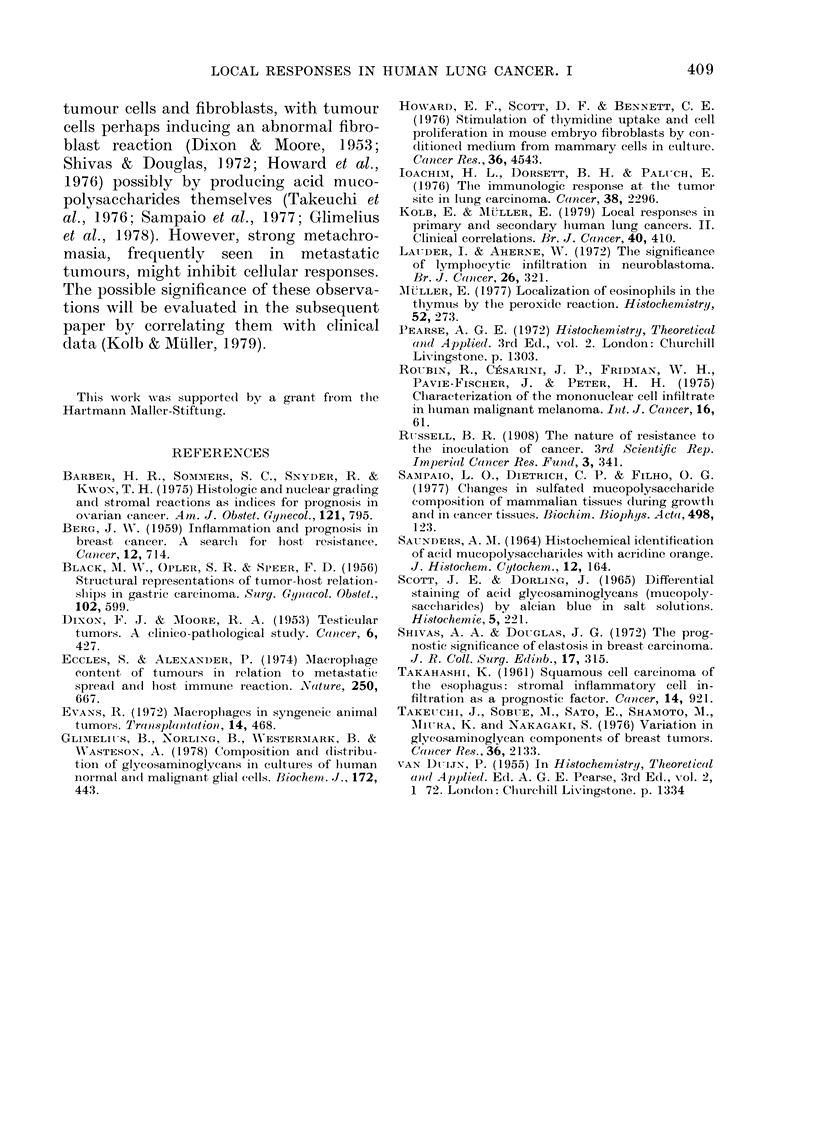# Local responses in primary and secondary human lung cancers. I. Patterns of cellular (eosinophils and macrophages) and extracellular (acid mucopolysaccharide) reactions.

**DOI:** 10.1038/bjc.1979.195

**Published:** 1979-09

**Authors:** E. Müller, E. Kolb

## Abstract

**Images:**


*Br. J. Cancer* (1979) **40**, 403

### LOCAL RESPONSES IN PRIMARY AND SECONDARY HUMAN LUNG CANCERS. I. PATTERNS OF CELLULAR (EOSINOPHILS AND MACROPHAGES) AND EXTRACELLULAR (ACID MUCOPOLYSACCHARIDE) REACTIONS

E. MÜLLER* AND E. KOLB†

*From the *Institute of Anatomy, University of Zürich, and the †Department of Surgery A, University Hospital, Zürich*

Received 6 April 1979 Accepted 30 May 1979

**Summary.—Local free cell (eosinophils and macrophages) and extracellular (acid mucopolysaccharides) reactions were studied by histochemical techniques in 72 primary lung cancers (Group A), 17 pulmonary metastases and 5 lung tumours of unknown origin (Group B). Strong cellular reactions were more frequent in Group A than in Group B, whereas extracellular reactions were more frequent in Group B than in Group A. In both groups the degrees of eosinophilic infiltration and accumulation of acid mucopolysaccharides tended to be negatively correlated. In contrast, macrophages and eosinophils showed a positive correlation in the primary cancers, but none in the other tumours.**

**The extracellular material had physical and chemical properties (solubility, affinity to stains and degradability by enzymes) of the sulphated acid mucopolysaccharides. From its distribution it appeared to be derived from fibroblasts and/or tumour cells.**

In some experimental tumour systems the host develops an immune reaction against a neoplastic transplant, mediated in part by infiltrating cells in contact with the tumour cells. Important effectors of this defence reaction appear to be macrophages (Evans, 1972; Eccles & Alexander, 1974). On the other hand, the concept of "stromal reaction", an ill-defined term coined by Russell (1908) has emerged from time to time for various observations in human tumour pathology: for "inflammatory infiltrates" in breast cancer (Berg, 1959) and carcinoma of the oesophagus (Takahashi, 1961) "elastosis" in breast carcinoma (Shivas & Douglas, 1972) "lymphocytic infiltrations" in gastric carcinoma (Black *et al.*, 1956), neuroblastoma (Lauder & Aherne, 1972) and ovarian cancer (Barber *et al.*, 1975), macrophages in melanomas (Roubin *et al.*, 1975) and plasma cells and antibody in lung cancer (Ioachim *et al.*, 1976). We now add another triad of pieces to the puzzle: eosinophils, macrophages and acid mucopolysaccharides in primary and secondary human lung cancers. Initially we were stimulated by the experimental "macrophage wave" but decided to focus on macrophages and eosinophils in the hope of gaining additional insight into the mechanisms of the host response. In the end the spectrum was widened by the inclusion of extracellular acid mucopolysaccharides. The choice of this parameter grew from accidental observations of odd strands of poorly structured metachromatic material between tumour cell groups and individual cells. No connection between the 3 parameters was implied at the beginning. A comparison between the results in 72 primary lung cancers, 17 pulmonary meta-

Correspondence to: Dr Edith Kolb, Universitätsspital Zürich, Chirurgische Klinik A, Rämistrasse 100, CH-8091 Zürich.

stases and 5 tumours of unknown origin is presented.

### MATERIALS AND METHODS

*Handling of tumour specimens.*—The tumours were processed immediately after they had been removed. Small pieces were cut from the margin of the neoplasm together with a rim of lung tissue, packed air tight, frozen and stored at −20°C. Care was taken to choose the material from intact, non-necrotic parts. At weekly intervals 4-6 tumours were worked up together by histochemical procedures and examined by the same person (E.M.).

*Staining techniques.*—The frozen tissues were cut at 8 $\mu$m in the cryostat and the sections dried at 4°C before incubation for histochemical reactions. For identification of the cell types studied, an appropriate enzyme-staining technique was used.

Eosinophils were demonstrated by the benzidine method (van Duijn, 1955) with addition of L-DOPA (Müller, 1977) for peroxidase (E.C. 1.11.1.7) whilst macrophages were identified with the $\alpha$-naphthyl-acetate method (Pearse, 1972) for non-specific esterases (E.C. 3.1.1.1).

For demonstration of non-specific metachromasia of mucopolysaccharides, toluidine blue and Giemsa-May-Grünwald staining were used.

For differential staining of acid glycosaminoglycans the alcian blue (Scott & Dorling, 1965) and the acridine orange-CTAC (Saunders, 1964) methods were used, each with the proper critical electrolyte concentrations.

### RESULTS

The histochemical observations were recorded without knowledge of operative or other clinical data, and before the histological diagnosis was known. Initially the tumours were studied for infiltrating cells only, either eosinophils or macrophages or both. However, it became evident after 39 tumours had been examined, that "metachromasia" observed with toluidine blue staining might also be worth systematic evaluation, because it was so conspicuous in some, but absent in other cases. Therefore, metachromasia was added as a third parameter in the subsequent 92 tumours examined and herein presented. The incomplete results of the first 39 tumours were omitted.

### *Eosinophils*

These cells, which contain peroxidase in abundance, were easy to identify by the brown-black reaction deposit of the enzyme developed histochemically. As Fig. 1 shows, the cells were spread singly or in clusters in the scarce stroma of tumours with a compact structure. On the other hand, in tumours growing more invasively into the parenchyma of the lung, the eosinophils were more irregularly scattered and mostly localized in the interalveolar septa. In addition, the blood vessels nearby sometimes contained numerous eosinophils. Occasionally the adjacent parenchyma of the lung showed small groups of lymphocytes arranged in pseudofollicles, sometimes surrounded by eosinophils.

### *Macrophages*

As Fig. 3 shows, these cells were identified by the reaction deposit for non-specific esterases. Not every macrophage showed equally strong enzyme activity; all shades of intensities being observed (Fig. 4). The macrophages were distributed in densely packed clusters within septa and alveoli throughout the parenchyma of the lung. They were, however, never detected within the venules surrounding the tumour.

### *Metachromasia*

Some tumours, when stained with alcian blue or toluidine blue, were interwoven with intensely metachromatic strands (Fig. 5) containing few cellular elements (Fig. 7). At higher magnification much metachromatic material could often be detected also between the tumour cells themselves (Fig. 7). Alcian blue staining with the addition of different concentrations of magnesium chloride, allowed partial characterization of the mucopolysaccharides in the strands. At low concentrations of $MgCl_2$ (0·1–0·3M) the ground

FIG. 1.—Eosinophils are demonstrated as dark dots, a reaction product for peroxidase. Note the distribution of the reactive cells within the surrounding stroma of a compact tumour. Counterstaining with toluidine blue.

FIG. 2.—Irregular distribution of eosinophils in an invasively growing lung tumour. Most of the reactive cells seem to be localized in the interalveolar septa. Peroxidase incubation and toluidine blue counterstaining.

FIG. 3.—Dense clusters of macrophages within the pulmonary alveoli and septa after incubation for non-specific esterases. The tissue of the invasively growing tumour (right corner) and the lung parenchyma are unstained.

FIG. 4.—Higher magnification of a macrophage cluster in the same lung tumour incubated for non-specific esterases. Note the varying enzyme intensities of the cells. No counterstaining.

FIG. 5.—Lung tumour with strands containing metachromatic ground-substance. Alcian blue staining at 0·1M $MgCl_2$.

FIG. 6.—Another section from the same tumour with alcian blue staining at 0·6M $MgCl_2$. tu = tumour, me = metachromasia, nc = necrosis.

FIG. 7.—Metachromasia shown with toluidine blue staining. Note the low cellular content within the strand. Small arrows indicate metachromatic material between the tumour cells.

substance of the strands showed no staining, but the tumour cells were reactive (Fig. 5). With increasing concentrations of $MgCl_2$ (0·45, 0·6, 0·8M) the ground substance became more and more reactive, while the tumour cells lost the stain (Fig. 6). From these results the metachromatic material could be chondroitin sulphate, heparin or keratin sulphate, but not hyaluronic acid. Confirming results were obtained with the acridine orange—CTAC-method.

Enzymic digestion studies showed the material to be resistant to hyaluronidase, neuraminidase, collagenase and trypsin; however, it could be washed out after digestion with pepsin. Therefore it appeared to be firmly bound to a protein backbone. Preliminary preparative extraction and electrophoresis (unpublished) showed that the metachromatic material had an electrophoretic mobility of its own, contained a small fraction of hyaluronidase-digestible, but a major non-digestible fraction. This fraction was also resistant to neuraminidase. Its electrophoretic mobility was distinct from extracted acid mucopolysaccharides of normal lung, trachea, cartilage, bronchial mucosa and peritracheal connective tissue used as controls, and from the markers commercial animal hyaluronic acid, chondroitin sulphates A and C and heparin. From these properties the material seemed to belong to the group of chondroitin sulphates B.

*Grading of cellular and extracellular reactions*

The following grading scales for the 3 parameters were used in the preliminary studies: (0), (+), (+ +) and (+ + +). In the definitive studies presented in this report, the degrees (+ +) and (+ + +) were no longer differentiated. They were mentally combined and are designated (+ + +) in the figures, meaning "strong reactivity" as opposed to "weak reactivity (+)" and "no reactivity (0)". In Figs. 8–10 the reactions were correlated separately for the primary lung cancers and all the other tumours taken together. Shaded areas were used to point out the main parameter under consideration and its major relationship to one other parameter. Fig. 8 shows the correlations

Fig. 8.—Correlation between macrophage and eosinophil infiltration in 70 primary lung cancers (A) and 22 metastatic and other lung tumours (B). For shaded areas see p. 406.

Fig. 9.—Correlation between metachromasia and eosinophil infiltration in 72 primary lung cancers (A) and 22 metastatic and other lung tumours (B). For shaded areas see p. 406.

between macrophages and eosinophils. Numerous macrophages were observed in 45/70 primary lung cancers (A); in 30/45 they were accompanied by numerous eosinophils, in 11/45 by few, and in only 4/45 were there no eosinophils. No correlation between the 2 cell types was evident however in the other tumours (B). In contrast, an inverse correlation of a similar degree was found between metachromasia and eosinophils (Fig. 9). No metachromasia was found in 38/72 primary tumours (A); this was associated with strong eosinophilia in 24/38, few eosinophils in 12/38 and none in 2/38 cases. There was also an inverse correlation

FIG. 10.—Correlation between metachromasia and macrophage infiltration in 70 primary lung cancers (A) and 22 metastatic and other lung tumours (B). For shaded areas see p. 406.

between the 2 parameters in the other tumours (B). Likewise, the correlation between metachromasia and macrophages, as demonstrated in Fig. 10, tended to be negative in the primary lung cancers (A), but was not clear-cut for the other group (B).

### DISCUSSION

The identification of reactive cell types in human tumours is often neglected, mainly because the relevant enzymes are destroyed by fixation, the usual staining is inadequate for this purpose and surgeons are more interested in histology than in immunology. The results presented show that more than half of the primary lung cancers induced a strong infiltration by free cells, usually both macrophages and eosinophils, and only about a third showed strong metachromasia; on the other hand, locally strong eosinophilia was found in only 5/22 other tumours, but strong metachromasia in 10 of them. Thus, the 2 tumour groups showed opposite tendencies for the cellular and extracellular parameters, and within the same group, the 2 parameters seemed to be inversely correlated. However, strong metachromasia, present in 20 primary tumours, was not incompatible with strong cellular reactivity; about half of these tumours showed strong intensity for both free cells and metachromasia. Of the other tumours, 2/22 showed this type of reaction. These observations indicate: that patterns of local response can be discerned in malignant lung tumours; that cellular and extracellular reactions may be correlated; and that there are differences between primary cancers (53 squamous-cell types, 9 undifferentiated tumours excluding oat-cell types, 7 adenocarcinomas and 3 alveolar-cell carcinomas; for further details see Kolb & Müller, 1979) and metastatic tumours or tumours of unknown origin (11 different histologies, including 6 hypernephromas and 5 teratomas). From the frequent pattern in primary tumours "strong cellular reactivity, no metachromasia" and the less frequent, but not to be ignored combination "strong cellular reaction, strong metachromasia", we suggest that the cellular and extracellular responses do not depend directly upon each other, but that the former expresses host responsiveness, while the latter expresses a complex interaction of tumour cells and fibroblasts, with tumour cells perhaps inducing an abnormal fibroblast reaction (Dixon & Moore, 1953; Shivas & Douglas, 1972; Howard *et al.*, 1976) possibly by producing acid mucopolysaccharides themselves (Takeuchi *et al.*, 1976; Sampaio *et al.*, 1977; Glimelius *et al.*, 1978). However, strong metachromasia, frequently seen in metastatic tumours, might inhibit cellular responses. The possible significance of these observations will be evaluated in the subsequent paper by correlating them with clinical data (Kolb & Müller, 1979).

This work was supported by a grant from the Hartmann Maller-Stiftung.

### REFERENCES

BARBER, H. R., SOMMERS, S. C., SNYDER, R. & KWON, T. H. (1975) Histologic and nuclear grading and stromal reactions as indices for prognosis in ovarian cancer. *Am. J. Obstet. Gynecol.*, **121**, 795.

BERG, J. W. (1959) Inflammation and prognosis in breast cancer. A search for host resistance. *Cancer*, **12**, 714.

BLACK, M. W., OPLER, S. R. & SPEER, F. D. (1956) Structural representations of tumor-host relationships in gastric carcinoma. *Surg. Gynacol. Obstet.*, **102**, 599.

DIXON, F. J. & MOORE, R. A. (1953) Testicular tumors. A clinico-pathological study. *Cancer*, **6**, 427.

ECCLES, S. & ALEXANDER, P. (1974) Macrophage content of tumours in relation to metastatic spread and host immune reaction. *Nature*, **250**, 667.

EVANS, R. (1972) Macrophages in syngeneic animal tumors. *Transplantation*, **14**, 468.

GLIMELIUS, B., NORLING, B., WESTERMARK, B. & WASTESON, A. (1978) Composition and distribution of glycosaminoglycans in cultures of human normal and malignant glial cells. *Biochem. J.*, **172**, 443.

HOWARD, E. F., SCOTT, D. F. & BENNETT, C. E. (1976) Stimulation of thymidine uptake and cell proliferation in mouse embryo fibroblasts by conditioned medium from mammary cells in culture. *Cancer Res.*, **36**, 4543.

IOACHIM, H. L., DORSETT, B. H. & PALUCH, E. (1976) The immunologic response at the tumor site in lung carcinoma. *Cancer*, **38**, 2296.

KOLB, E. & MÜLLER, E. (1979) Local responses in primary and secondary human lung cancers. II. Clinical correlations. *Br. J. Cancer*, **40**, 410.

LAUDER, I. & AHERNE, W. (1972) The significance of lymphocytic infiltration in neuroblastoma. *Br. J. Cancer*, **26**, 321.

MÜLLER, E. (1977) Localization of eosinophils in the thymus by the peroxide reaction. *Histochemistry*, **52**, 273.

PEARSE, A. G. E. (1972) *Histochemistry, Theoretical and Applied.* 3rd Ed., vol. 2. London: Churchill Livingstone. p. 1303.

ROUBIN, R., CÉSARINI, J. P., FRIDMAN, W. H., PAVIE-FISCHER, J. & PETER, H. H. (1975) Characterization of the mononuclear cell infiltrate in human malignant melanoma. *Int. J. Cancer*, **16**, 61.

RUSSELL, B. R. (1908) The nature of resistance to the inoculation of cancer. *3rd Scientific Rep. Imperial Cancer Res. Fund*, **3**, 341.

SAMPAIO, L. O., DIETRICH, C. P. & FILHO, O. G. (1977) Changes in sulfated mucopolysaccharide composition of mammalian tissues during growth and in cancer tissues. *Biochim. Biophys. Acta*, **498**, 123.

SAUNDERS, A. M. (1964) Histochemical identification of acid mucopolysaccharides with acridine orange. *J. Histochem. Cytochem.*, **12**, 164.

SCOTT, J. E. & DORLING, J. (1965) Differential staining of acid glycosaminoglycans (mucopolysaccharides) by alcian blue in salt solutions. *Histochemie*, **5**, 221.

SHIVAS, A. A. & DOUGLAS, J. G. (1972) The prognostic significance of elastosis in breast carcinoma. *J. R. Coll. Surg. Edinb.*, **17**, 315.

TAKAHASHI, K. (1961) Squamous cell carcinoma of the esophagus: stromal inflammatory cell infiltration as a prognostic factor. *Cancer*, **14**, 921.

TAKEUCHI, J., SOBUE, M., SATO, E., SHAMOTO, M., MIURA, K. and NAKAGAKI, S. (1976) Variation in glycosaminoglycan components of breast tumors. *Cancer Res.*, **36**, 2133.

VAN DUIJN, P. (1955) In *Histochemistry, Theoretical and Applied.* Ed. A. G. E. Pearse, 3rd Ed., vol. 2, 1 72. London: Churchill Livingstone. p. 1334